# Mechanical forces regulate the interactions of fibronectin and collagen I in extracellular matrix

**DOI:** 10.1038/ncomms9026

**Published:** 2015-08-14

**Authors:** Kristopher E. Kubow, Radmila Vukmirovic, Lin Zhe, Enrico Klotzsch, Michael L. Smith, Delphine Gourdon, Sheila Luna, Viola Vogel

**Affiliations:** 1 Department of Biology, James Madison University, Harrisonburg, Virginia 22807, USA; 2 Department of Health Sciences and Technology, ETH Zurich, CH-8093 Zürich, Switzerland; 3 Centre for Vascular Research, ARC Centre of Excellence in Advanced Molecular Imaging and Australian Centre for Nanomedicine, University of New South Wales, Sydney, New South Wales 2052, Australia; 4 Department of Biomedical Engineering, Boston University, Boston, Massachusetts 02215, USA; 5 Department of Material Science and Engineering, Cornell University, Ithaca, New York 14853, USA

## Abstract

Despite the crucial role of extracellular matrix (ECM) in directing cell fate in healthy and diseased tissues—particularly in development, wound healing, tissue regeneration and cancer—the mechanisms that direct the assembly and regulate hierarchical architectures of ECM are poorly understood. Collagen I matrix assembly *in vivo* requires active fibronectin (Fn) fibrillogenesis by cells. Here we exploit Fn-FRET probes as mechanical strain sensors and demonstrate that collagen I fibres preferentially co-localize with more-relaxed Fn fibrils in the ECM of fibroblasts in cell culture. Fibre stretch-assay studies reveal that collagen I’s Fn-binding domain is responsible for the mechano-regulated interaction. Furthermore, we show that Fn-collagen interactions are reciprocal: relaxed Fn fibrils act as multivalent templates for collagen assembly, but once assembled, collagen fibres shield Fn fibres from being stretched by cellular traction forces. Thus, in addition to the well-recognized, force-regulated, cell-matrix interactions, forces also tune the interactions between different structural ECM components.

## Abstract (web summary)

Assembly of a collagen extracellular matrix requires the presence of fibronectin, but the mechanisms that direct this assembly are not known. Here the authors show that collagen I uses relaxed fibronectin fibrils as a template for assembly, and in turn shield fibronectin fibrils from force-mediated stretching.

## Graphical abstract

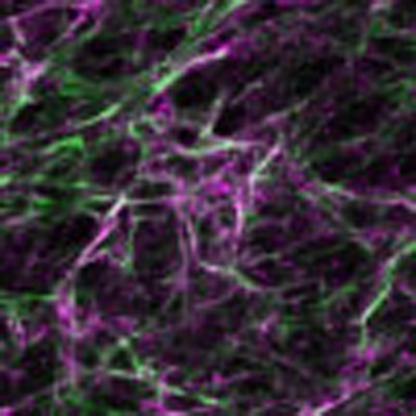

The assembly, composition and hierarchical architecture of extracellular matrix (ECM) tightly controls early tissue development^1^, cancer progression^2^,^3^,^4^,^5^,^6^, as well as wound healing and the progression of many diseases associated with fibrosis^7^,^8^,^9^,^10^. Embryonic development and normal wound healing are characterized by the sequential deposition of multiple ECM proteins into a complex, ordered structure of interwoven fibrils^1^,^7^. In particular, both fibronectin (Fn) and collagens are needed for normal embryo development^1^,^11^. Similarly, in wound healing, the first matrix actively assembled by fibroblasts is made of Fn, which gradually replaces the provisional fibrin clot^7^,^8^,^9^. Over time, fibroblasts then deposit a composite Fn and collagen matrix (granulation tissue) and the wound is ultimately remodelled into a predominately collagen-based ECM. The architecture of the collagen matrix is therefore central to tissue function, and deviations can lead to biomaterial encapsulation^12^, fibrotic diseases, and stromagenesis^8^,^13^. Fn and collagens are thus two prominent structural ECM constituents in development, wound healing and cancer. Despite the known spatiotemporal coordination between Fn and collagen matrices, most *in vitro* studies of cell-ECM interactions have used model systems that involve either Fn or collagen, and none to our knowledge have studied whether Fn-collagen interactions are mechano-regulated. Finally, it is important to note that in all of the above processes, cell-generated mechanical stresses of varying magnitude are exerted on ECM fibres^1^,^14^,^15^, for example, by myofibroblasts in the granulation tissue^16^.

Cell-mediated Fn fibril assembly itself is known to be mechano-regulated^17^. Cell-generated traction forces are sufficient to stretch Fn ECM fibres several fold^18^,^19^,^20^ and mechanically unfold at least some of Fn’s more than 54 modules^21^,^22^,^23^,^24^. Stretch-induced alteration of Fn’s secondary structure changes Fn’s interactions with other Fn-binding proteins (Fig. 1). For example, a two-to-three-fold stretching of Fn fibrils, as routinely seen in fibroblast cell cultures^23^, increases the exposure of non-disulfide-bonded cysteines buried in FnIII modules^22^,^24^, alters Fn–Fn binding^17^,^19^, increases the non-specific binding of albumin and casein^25^, and destroys N-terminal bacterial binding sites^26^,^27^. Interestingly, while monomeric collagen I can be induced in the laboratory to polymerize via an entropy-driven process, its assembly *in vivo* is cell-mediated and requires, in particular, active Fn fibrillogenesis^11^,^28^ (although one instance of Fn-independent collagen fibrillogenesis has been published^29^). The specific binding of collagen type I’s α1 chain to Fn’s gelatin-binding domain (Fig. 1) is necessary for collagen’s initial deposition^30^,^31^. In cell culture, exogenously added soluble collagen I is known to organize into matrix fibres that co-localize with pre-existing Fn fibrils^32^,^33^. These interactions involve collagen binding sites that are located on Fn’s N-terminus (Fig. 1) on modules FnI_6_, FnII_1-2_ and FnI_7-9_(refs 34, 35, 36).

Since the mechanism by which Fn fibrils direct the deposition of collagen fibres is not known, in this study we asked whether the mechanical strain of the Fn matrix fibrils assembled and held under tension by fibroblasts guides collagen I matrix assembly. To study the functional interplay of Fn and collagen I in ECM, we added Förster resonance energy transfer (FRET)-labelled Fn (Fn-FRET) in small quantities into the cell culture medium such that NIH 3T3 fibroblasts could collect it together with unlabelled plasma Fn^21^,^23^ and incorporate it into their own ECMs^37^. Fn-FRET thus acts as an *in situ* probe for Fn fibre strain (extension). The production of collagen I matrix was stimulated by adding ascorbic acid to the medium^38^. Ascorbic acid is a cofactor for the enzyme prolyl-4-hydroxylase, which is required to convert procollagen’s proline residues to hydroxyproline; in its absence, most collagen fibre synthesis is prevented^39^. Measuring intramolecular FRET in Fn matrices with and without collagen allowed us to observe how Fn conformation and fibre strain change as a function of time and matrix composition^23^,^40^. Co-localization analysis of Fn-FRET and immunostained collagen I fibres allowed us to ask whether collagen preferentially co-localizes with stretched or relaxed Fn ECM fibres. Finally, we investigated the possible mechanism of our *in situ* observations by using a stretch assay^19^,^26^ to address whether Fn fibril strain alters the binding of collagen I’s Fn-binding domain.

Our results show that fibroblasts construct the ECM network by initially depositing Fn fibres, followed by collagen I fibres, which interact preferentially with relaxed Fn in the ECM. The presence of collagen I fibres and their specific molecular interaction with Fn limit the ability of fibroblasts to stretch and mechanically unfold Fn fibres. As the collagen I content in cell-derived ECMs increases, collagen fibres supersede Fn fibres the primary tension-bearing matrix elements. We thus identify a reciprocal mechano-regulation in ECM in which Fn conformation initially guides the assembly of collagen I fibres by directing its co-localization; but later, mature collagen fibres stabilize Fn fibres against cell-generated tensile forces.

## Results

### Ascorbic acid upregulates collagen I deposition in Fn ECM

NIH 3T3 fibroblasts were seeded on Fn-coated glass in medium with or without ascorbic acid, and supplemented with Fn-FRET. Over the first 24 h, the fibroblasts cultured in the presence of ascorbic acid (Fig 2g–i) produced collagen fibres that co-localized with Fn fibrils. Cells cultured without ascorbic acid produced comparatively little collagen matrix (Fig 2a–c). This difference was more pronounced after 72 h in culture (Day 3): both cultures showed increased Fn matrix deposition (Fig. 2d,j), but only the culture with ascorbic acid exhibited a concurrent increase in collagen matrix (Fig. 2 compare d–f and j–l). Ascorbic-acid-supplemented cultures also showed elevated Fn matrix deposition relative to untreated cultures, suggesting that although Fn is required for collagen fibre formation^11^, collagen I, to some degree, enhances Fn matrix formation^41^.

These observations agree with previous work showing that NIH 3T3 fibroblasts produce few collagen fibres when cultured up to 20 days in serum-containing medium^42^, but upregulate collagen I deposition within the first 12 h when the medium is supplemented with ascorbic acid^38^. Some collagen can be deposited in the absence of supplemental ascorbic acid (Fig. 2b,e)^38^,^43^; however collagen that lacks hydroxyproline residues has a lower stability and melting temperature owing to inefficient packing within fibres^39^. Thus collagen I fibrillogenesis within early, cell-generated ECMs can be upregulated through supplementation of ascorbic acid in the culture medium.

### Cell stretching of Fn is reduced in Fn-collagen ECMs

Significant forces are exerted by cells during their migration through and remodelling of 3D ECM^44^, which lead to matrix fibre rearrangements^4^,^15^ and gradually increasing Fn strains and conformational unfolding^21^,^23^,^40^,^45^,^46^,^47^. Because collagen I fibers are much stiffer than Fn fibrils^22^,^48^, we asked whether the co-deposition of collagen and Fn fibrils affected the cell-mediated stretching of Fn fibrils. We repeated the above experiments—culturing cells for 3 days with or without ascorbic acid—but supplemented the medium with Fn labelled with donor and acceptor fluorophores (Fn-FRET)^23^,^40^. The cells incorporated the Fn-FRET into their ECM fibres. Since our FRET-labelled Fn carried many donors and acceptors, changes in intramolecular FRET ratios, measured as peak donor and acceptor fluorescence intensities via spectral confocal microscopy, were assayed as an indicator of Fn’s molecular conformation^23^ and fibril strain^19^. Furthermore, supplementing the medium with an excess of unlabelled Fn (also incorporated into fibres) prevented FRET from occurring between adjacent molecules so that only intramolecular FRET was measured^23^,^49^.

**Figure 1:**
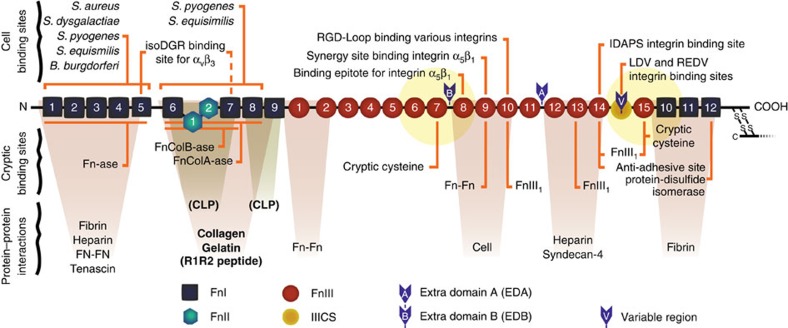
Fn’s major cell and ECM-protein binding sites. Diagram of a monomer of plasma Fn showing selected binding and interaction sites (adapted from^45^). Fn exists as a dimer of two nearly identical monomers, linked at the C-terminus by disulfide bonds. The yellow circles show the 6 nm Forster energy transfer radius for the acceptor dyes that are located on modules FnIII_7_ and FnIII_15_ in FRET-labelled Fn. While the FRET radii are drawn roughly to scale to indicate which modules are covered, other aspects of the diagram are not necessarily to scale. The canonical collagen/gelatin-binding domain, the R1R2 bacterial-adhesin-derived peptide^52^, and the two binding sites for a CLP encompassing the Fn-binding domain of the collagen I α_1_ chain^35^,^36^ are shown in bold.

**Figure 2:**
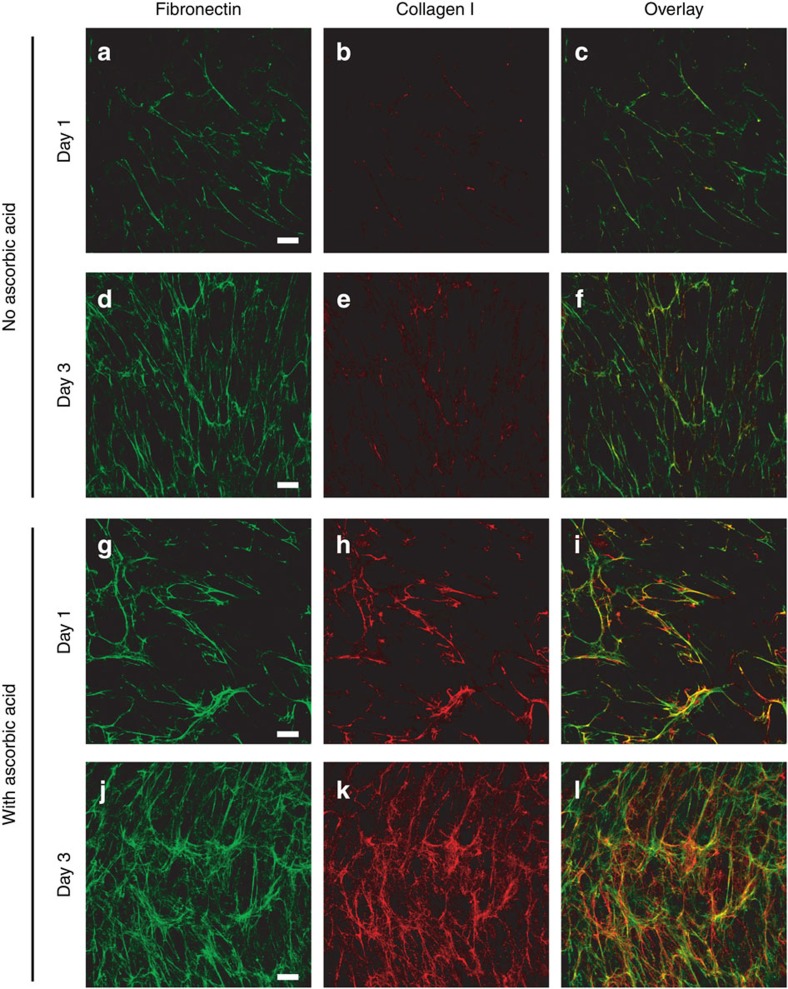
Cells receiving supplemental ascorbic acid upregulate collagen matrix assembly. NIH 3T3 fibroblasts were seeded on Fn-coated glass with medium containing fluorescently labelled Fn (Alexa 488) in the absence (**a**–**f**) or presence (**g**–**l**) of 50 μg ml^−1^ ascorbic acid, and were cultured for 1 or 3 days, then fixed and immunostained for collagen type I. Collagen matrix production was greatly reduced in the absence of supplemental ascorbic acid. Images are z-projections of representative confocal z-stacks selected from five independent experiments. The third column of images shows the Fn (green) and collagen (red) images overlaid. Bar=20 μm.

FRET was calibrated in solutions of specific concentrations of the chemical denaturant guanidine hydrochloride (GdnHCl; Supplementary Table 1)^23^,^40^. Fn begins to lose its secondary structure in concentrations of GdnHCl >1 M and is completely denatured in 4 M GdnHCl^23^,^49^. The 1 M GdnHCl denaturation curve point was measured using Fn that had been reduced to monomers with dithiothreitol (DTT) to prevent additional FRET caused by crossover of the Fn-dimer arms. The FRET ratio measured at 1 M GdnHCl also correlates with the FRET ratio below which we see a large increase in Fn unfolding and exposure of otherwise cryptic cysteines (Fig. 1) in Fn fibres (∼150–200% strain or 2.5–3 × extension)^22^. FRET ratios for monomeric Fn in 1 M GdnHCl (DTT-treated) and for dimeric Fn in 4 M GdnHCl buffer solutions are provided as benchmarks on all plots of ECM FRET data. While each set of experiments is internally consistent in terms of shifts of FRET histograms, because of small batch-to-batch differences in Fn-FRET fluorophore labelling (Supplementary Table 1), comparisons between experiments utilizing different Fn-FRET batches should be made using these benchmarks.

**Figure 3:**
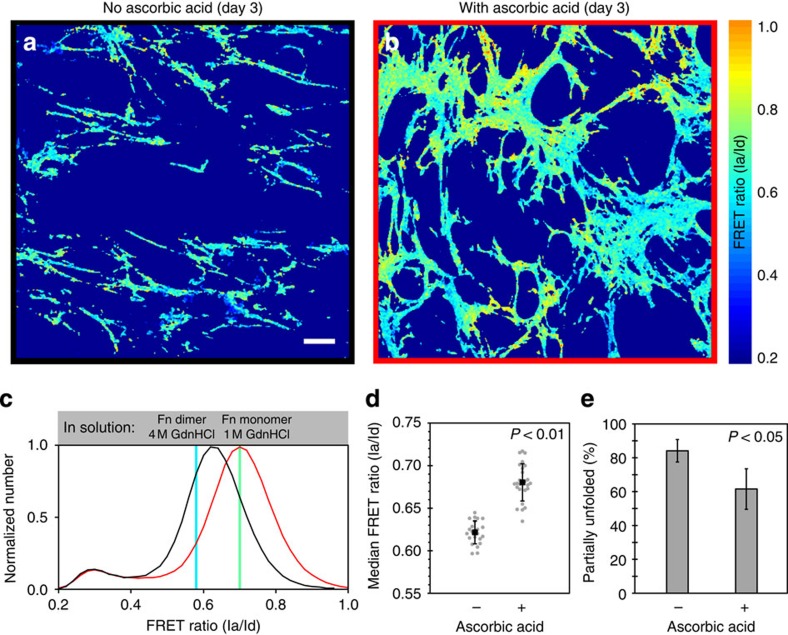
Ascorbic-acid-treated cell cultures produce less-strained Fn matrix fibres. NIH 3T3 fibroblasts were allowed to incorporate small amounts of Fn-FRET into their matrices for 3 days in the absence (**a**) or presence (**b**) of 50 μg ml^−1^ ascorbic acid. (**a**–**b**) Single z-slice images from the specified sample, colour-coded to reflect the calculated FRET ratios (see colour bar on right). (**c**) Representative normalized histograms of the FRET ratio distributions compiled from eight measurements for each treatment from a single experiment (black, no ascorbic acid; red, with ascorbic acid). The Fn-FRET denaturation points for the Fn monomer in 1 M GdnHCl and for the Fn dimer in 4 M GdnHCl points (see Supplementary Table 1) are indicated with coloured, vertical lines. Fn fibres with FRET ratios less than at the 1 M GnHCl calibration point (for Fn in solution) indicate partial secondary/tertiary structure loss due to unfolding. (**d**) Median FRET ratios from four independent experiments. Grey points represent individual measurements (23 for samples without and 25 for samples with ascorbic acid); black squares represent means (error bars, s.d.). (**e**) The same data as in panel (**d**) plotted as the mean (±s.d.) per cent unfolding of each group. Per cent partial Fn unfolding is the percentage of pixels with FRET ratios less than that seen at the 1 M denaturation curve point. Samples with and without ascorbic acid had significantly different median FRET ratios (*P*<0.01) and mean per cent unfolding values (*P*<0.05) (ANOVA, followed by a Tukey–Kramer *post-hoc* test were used because these data were analysed with additional groups shown in Fig. 4). Bar=20 μm.

FRET ratios were determined pixel-by-pixel for 3-day ECMs produced in the absence or presence of ascorbic acid. Figure 3a,b show single, representative z-slices from these samples that have been colour-coded based on FRET ratios (see colour bar). Each FRET ratio histogram (Fig. 3c) shows data from eight z-stacks for each condition, from a single representative experiment. The small secondary peak centred at a FRET ratio of 0.3 corresponds to small amounts of Fn-FRET that have been endocytosed by the cells as previously described^46^. Surprisingly, Fn ECM fibrils assembled by fibroblasts cultured in the absence of ascorbic acid were more stretched on average than those formed in the presence of ascorbic acid as reflected by their lower FRET ratio distribution (Fig. 3c). Over multiple experiments, median FRET ratios of samples supplemented with ascorbic acid were significantly higher (P<0.01; analysis of variance (ANOVA), Tukey-Kramer post-hoc test) than those without (Fig. 3d). This difference equated to 28% less Fn experiencing unfolding (that is, having a FRET ratio below the 1 M line) in the ascorbic-acid-treated ECMs (Fig. 3e). Increased collagen deposition is often associated with increased tissue stiffness and cell contractility^2^,^3^—conditions that, at least in a single-component, Fn ECM, support increased Fn strain through cell-mediated stretching^18^,^45^. Nevertheless, we observe that Fn fibrils in Fn-ECMs interlaced with collagen fibres are less stretched than in Fn-only ECMs.

### Collagen is the primary load-bearer in Fn-collagen ECM

**Figure 4:**
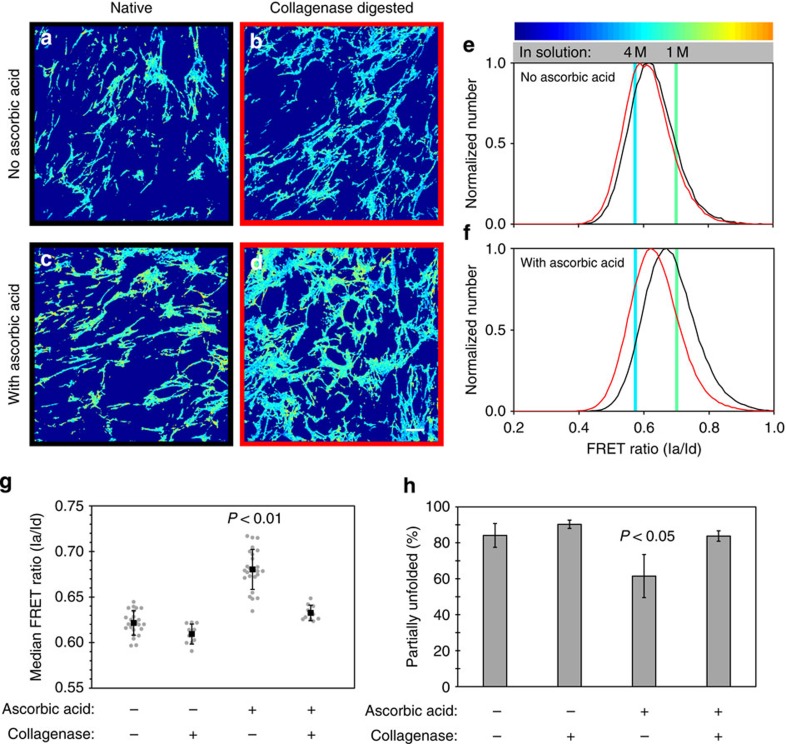
Selective digestion of collagen I fibres with collagenase increases Fn matrix strain in ECM produced by fibroblasts. Live, Fn-FRET-labelled, 3-day samples that were cultured in the absence (**a**,**b**,**e**) or presence (**c**,**d**,**f**) of 50 μg ml^−1^ ascorbic acid were digested with collagenase for 3 h (**b**,**d**) or kept undigested (‘native’, **a**,**c**). (**a**–**d**) Single z-slices from the indicated matrix that have been colour-coded to visualize the different FRET ratios (see colour bar). (**e**–**f**) Representative FRET ratio distributions of native (black) and digested (red) ECMs assembled by fibroblasts in the presence or absence of ascorbic acid in the medium, compiled from two independent experiments (data displayed as in Fig. 3). Note that native and collagenase-digested data were taken from separate samples. (**g**) Median FRET ratios compiled from multiple experiments (native samples, four experiments; digested samples, two experiments). Grey points represent individual measurements (from left to right, *n*=23, 10, 25 and 10); black squares represent means (error bars, s.d.). (**h**) The same data as in panel (**g**) plotted as the mean (±s.d.) per cent unfolding of each group. Samples supplemented with ascorbic acid, but not digested with collagenase had significantly different median FRET ratios (*P*<0.01) and mean partial unfolding percentages (*P*<0.05) than the other groups (ANOVA, Tukey–Kramer *post hoc*). Scale bar=20 μm.

To determine if the presence of collagen I fibres was in fact preventing cell-generated forces from stretching the co-deposited Fn fibrils, collagenase was added to 3-day-old, ascorbic-acid-supplemented cultures and the Fn-FRET ratios were measured after 3 h (Fig. 4). Collagenase treatment eliminated essentially all collagen fibres, as assessed by immunostaining (Supplementary Fig. 1), but did not affect the Fn matrix architecture, as previously shown^50^, since Fn does not contain any collagenase cleavage sites. In similar cultures, collagenase was shown not to affect cell viability^50^. Collagenase digestion resulted in an increase in Fn strain and unfolding in ascorbic-acid-supplemented samples, but had little effect on ECMs without collagen I content, as illustrated by the colour changes in Fig. 4a–d and the histogram shifts in Fig. 4e,f. While undigested ascorbic-acid-supplemented samples had significantly higher (*P*<0.01; ANOVA/Tukey–Kramer) median FRET ratios than control samples, this difference was eliminated on collagenase digestion (Fig. 4g). This change was reflected in a 36% increase in Fn fibres experiencing partial unfolding on digestion of the collagen in ascorbic-acid-supplemented samples (Fig. 4h). These data suggest that collagen fibres, once assembled, serve as the primary tension-bearing elements in these Fn/collagen composite ECMs. Fibroblasts can actively stretch Fn fibrils again, but only once collagen-Fn interactions are disrupted (Fig. 4).

### Collagen’s binding to Fn is important for its load-bearing

**Figure 5:**
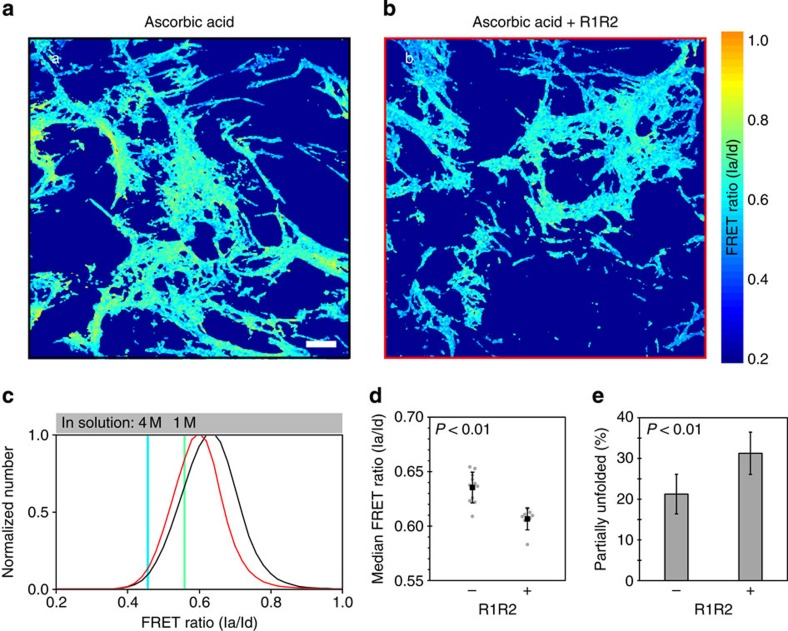
Inhibition of Fn-collagen I interactions with the R1R2 peptide increases Fn matrix fibre strain. Live, Fn-FRET-labelled, 3-day samples were cultured in the presence of 50 μg ml^−1^ ascorbic acid and with (**b**) or without (**a**) the inhibitory R1R2 peptide. (**a**–**b**) Single z-slices from the indicated matrix that have been colour-coded to indicate the FRET ratios (see colour bar). (**c**) Representative normalized histograms of the FRET ratio distributions compiled from three z-stacks for each treatment from a single experiment (black, control; red, with R1R2). (**d**) Median FRET ratios compiled from three independent experiments. Grey points represent individual measurements (11 for group without and 8 for group with R1R2); black squares represent means (error bars, s.d.). (**e**) The same data as in panel (**d**) plotted as the mean (±s.d.) per cent unfolding of each group. Samples with and without R1R2 had significantly different median FRET ratios (*P*<0.01) and mean partial unfolding percentages (*P*<0.01; Student’s *t*-test). R1R2 had no effect on FRET in the absence of collagen fibres in the ECM (see Supplementary Fig. 2). Scale bar=20 μm.

Fn and collagen I bind directly to each other^34^,^35^,^36^ and closely associate in the ECM^11^,^28^. We therefore asked whether the above results were due simply to the presence of collagen I in the ECM or to actual molecular binding interactions between Fn and collagen. We thus inhibited Fn-collagen binding using a previously reported interfering peptide, R1R2, which is derived from a bacterial adhesin and has been shown to block Fn binding to collagens I, II and III^51^,^52^. This peptide was added to the media of fibroblasts in addition to ascorbic acid and Fn-FRET, as in previous experiments, and the cells were cultured for 3 days. Cells treated with R1R2 produced Fn ECM with collagen fibres (Fig. 5); however the Fn fibres showed significantly (*P*<0.01; Student’s *t*-test) lower FRET ratios, relative to ECM produced in the presence of ascorbic acid but without R1R2 (Fig 5a–d). Treatment with R1R2 resulted in a 32% increase in Fn fibres with partial loss of secondary structure (Fig. 5e). R1R2 had no direct effect on the Fn-FRET of ECMs lacking collagen (Supplementary Fig. 2). These data show that partial inhibition of Fn-collagen interactions with R1R2, without eliminating collagen fibres from the ECM, increases Fn unfolding, suggesting that the molecular binding interaction is important for collagen’s load-bearing role in composite ECMs. A previous study demonstrated that R1R2 inhibited all collagen fibre deposition; however, that study used Fn-null fibroblasts that were totally reliant on exogenous Fn for matrix production^52^. There is strong evidence that Fn-collagen interactions can begin within the cell (that is, before secretion)^39^; the exogenous R1R2 peptide in our experiments is not inhibiting these initial interactions, which may account for the ability of our cells to assemble collagen fibres.

### Collagen fibres preferentially co-localize with relaxed Fn

**Figure 6:**
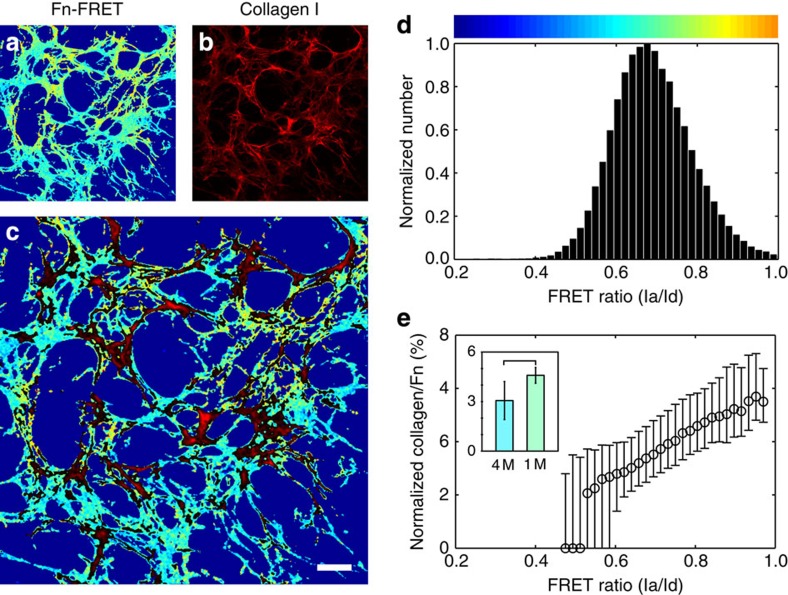
Collagen fibres in ECM preferentially co-localize with relaxed Fn fibres. Fibroblasts were cultured for 3 days with trace amounts of Fn-FRET and 50 μg ml^−1^ ascorbic acid in the medium. (**a**–**c**) Single z-slice images of the Fn-FRET (**a**; colour-coded as in Fig. 3), collagen I immunostaining (**b**, red) and overlay (**c**) are shown for a representative field-of-view. For clarity, the overlay image only shows collagen pixels (red) brighter than the median intensity. (**d**) Histogram of the FRET ratios from (**a**), presented as in Fig. 3. (**e**) Co-localization of collagen and Fn as a function of the FRET ratios. The ratio of collagen intensity to Fn-FRET ratio of each co-localized pixel in (**c**) was plotted as a function of the pixel’s respective FRET ratio; the entire plot was normalized to the area under the curve and expressed as a per cent. Data points are medians and error bars are the 25th and 75th percentiles; data are only reported for bins containing more than 50 pixels. The inset shows mean (±s.d.) collagen/Fn values for FRET ratios at the 1 M and 4 M denaturation curve points, taken from 23 measurements across four experiments. The two groups are significantly different (*P*<0.01, Student’s *t*-test). Bar=20 μm.

While our results point to a role for collagen fibres in regulating Fn strain and conformation in ECM, it is well established that collagen matrix deposition *in vitro* is guided by and dependent on active, concurrent Fn fibrillogenesis^11^. The molecular mechanisms behind this regulatory interaction though are poorly understood. Since many Fn-protein interactions are influenced by strain-dependent changes in Fn conformation^14^,^26^, we asked whether Fn conformation regulates collagen deposition. We previously observed Fn-strain-dependent co-localization of Fn and collagen fibres in simplified, pre-formed Fn or collagen matrices^33^,^47^. Here, we examined the effect of Fn strain on the *de novo* co-deposition of collagen using 3-day-old, ascorbic-acid-supplemented cultures containing Fn-FRET and immunostained for collagen I. The co-localization of Fn and collagen was quantified as a function of Fn fibre strain (FRET ratio). Collagen fibres preferentially co-localized with Fn fibrils with relatively high FRET ratios (Fig 6a–c), indicating low levels of fibre strain. The histogram of FRET ratios (Fig. 6d) and the corresponding normalized collagen intensities (Fig. 6e) from the representative field-of-view quantifies this trend, showing increasing collagen co-localization with increasing FRET ratios (decreasing strain). This trend is conserved across multiple experiments (Supplementary Fig. 3) and is summarized by the inset in Fig. 6e, which compares normalized collagen intensities associated with 1 M and 4 M FRET ratio points in solution. These data demonstrate that collagen co-localized preferentially with Fn fibrils in a relaxed conformation, that is, with fibrils that displayed FRET ratios corresponding to <3 × extension (200% strain)^22^ and to Fn in a 1 M GndHCl buffer solution.

### Fn conformation directs collagen-Fn binding

**Figure 7:**
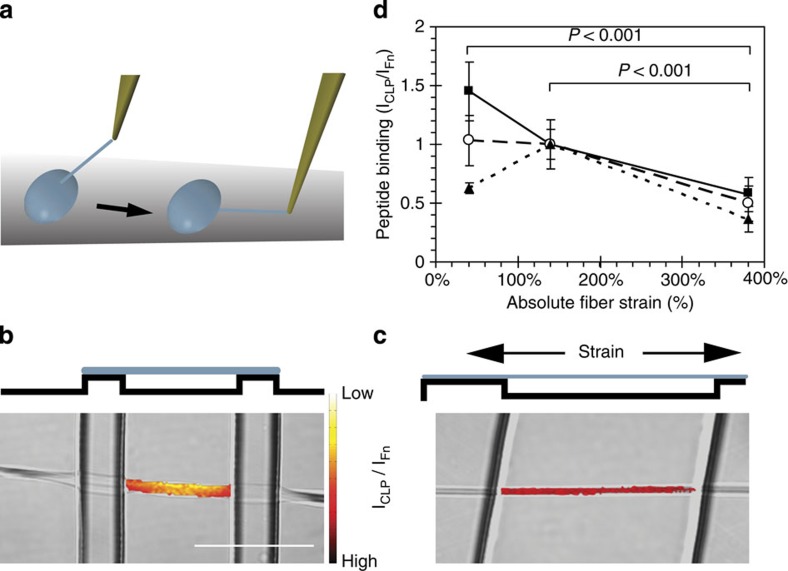
The Fn-binding synthetic collagen peptide (CLP) binds preferentially to relaxed Fn fibres in single Fn fibre stretch assays. (**a**) Cartoon showing the fabrication of manually deposited fibres (adapted from ref. 19). A peptide tip is immersed slowly in a concentrated solution of Fn, removed to initiate Fn fibrillogenesis, and the resulting Fn fibres are then deposited over micro-fabricated PDMS trenches. The elastic PDMS substrate is stretched or relaxed using a uniaxial mechanical straining device (52). Fn fibres labelled with Fn-Alexa647 and under different strains were incubated with an Alexa488-labelled CLP (corresponds to the Fn-binding region of collagen I). (**b**,**c**) The intensity of the CLP bound to the fibre was normalized by dividing by the Fn-Alexa647 intensity (see Materials and Methods section). The images show a side-view schematic of relaxed (**b**) and stretched (**c**) fibres freely suspended over the PDMS trenches. A top–down view (differential interference contrast) of a fibre crossing a trench, is overlaid with the CLP signal, colour-coded by the CLP/Fn intensity ratio. Note that only the CLP signal in the area suspended over the trench was analysed although CLP bound to the entire fibre. (**d**) The average intensity ratio (±s.d.) of CLP to Fn intensity, normalized to the mean value at the 140% strain point, is plotted versus absolute strain. Each line represents an independent experiment and each point represents measurements from at least six fibres. All 380% strain points are significantly different (*P*<0.001) than the 40 and 140% strain points from the same experiment (ANOVA, Tukey–Kramer *post hoc*). Bar=50 μm.

Since the presence of collagen matrix reduces global Fn fibre strain within the ECM (Figs 3 and 4), one explanation of the preferential co-localization of collagen and relaxed Fn fibrils (Fig. 6) is that the presence of collagen fibres mechanically stabilize Fn fibrils, thereby preventing Fn fibre stretching on a local level. In addition, the observed co-localization patterns could be the result of a conformation-dependent Fn-collagen interaction. Since co-localization at the resolution of pixels does not necessarily implicate molecular interactions, we directly tested whether Fn conformation regulates its binding to collagen I—a necessary prerequisite for collagen matrix assembly^30^. Because collagen self-assembles, even at very low concentrations^53^, we used a synthetic collagen peptide (CLP) derived from the Fn-binding region of the α_1_ chain of type I collagen (residues Q_778_-G_799_) ref. 35 and determined the level of its binding to single Fn fibrils with defined conformations and strains. The CLP was fluorescently labelled with Alexa 488 on an additional terminal cysteine. Fn fibres were manually pulled from droplets of concentrated Fn solution (only 10% was fluorescently labelled with Alexa 647 to prevent fluorescence quenching at low strain) and suspended over stretchable, micro-fabricated, PDMS trenches in a one-dimensional strain device (Fig. 7a)^19^,^22^,^54^. Manually pulled fibres are deposited across the trenches with a pre-strain of ∼140% (refs 19, 22), therefore the fibres were either relaxed or further strained to sample a broad range of strains typically seen in fibroblast-produced ECMs *in vitro*^19^,^23^,^26^,^46^. Ratiometric fluorescence intensity measurements of the CLP and Fn fibres were acquired (Fig 7b, c). Figure 7d shows CLP/Fn ratios for three independent experiments where the absolute strains have been normalized to the 140% strain point. Although there is variability in the 40% strain point, all three experiments show a significant overall decrease in CLP binding at high strains and an average 52% drop over the range of strains. In contrast, we have shown previously that non-specific binding of proteins to Fn is upregulated by stretching Fn fibres^25^ owing to the increased exposure of hydrophobic binding sites. The binding of the CLP is downregulated by stretching Fn fibres, thus suggesting that specific binding sites are being destroyed. Therefore, the association of Fn and collagen I can be modulated by conformational changes induced by cell-mediated mechanical stretching.

## Discussion

To gain insights into the mechanisms that enable the assembly of complex multi-component ECMs, we probed the tension-regulated interplay between Fn and collagen I fibres in ECM assembled by fibroblasts in cell culture. Using a FRET-based co-localization analysis in fibroblast-generated ECM (Fig. 6) and strain-dependent binding studies on manually deposited Fn fibres (Fig. 7), we demonstrated that collagen I fibres and the Fn-binding domain of collagen I interact preferentially with relaxed Fn fibres. Once assembled, collagen fibres restrict the ability of fibroblasts to further stretch and mechanically unfold Fn fibres (Figs 3, 4 and 5); instead, collagen fibres become the primary tension-bearing elements in the ECM. In 3-day ECMs, fibroblasts can only stretch Fn fibrils on inhibition of Fn-collagen binding interactions by the R1R2 peptide (Fig. 5) or by elimination of collagen I altogether (Fig. 4). Our findings thus reveal a reciprocal mechano-regulation among structural ECM proteins in which Fn conformation initially directs the hierarchical assembly of ECM by regulating collagen I co-localization; later, mature collagen fibres stabilize Fn conformation and fibre elongation against cell traction forces.

Studies have shown that the assembly of collagen I fibres requires the Fn’s collagen binding domain (Fig. 1): deleting modules I_6_ through I_9_ (ref. 52) or competing with Fn’s binding to collagen using an antibody to the same region^30^ both prevent collagen fibre assembly. Our results thus suggest a mechanism in which mechanical stretching of Fn perturbs Fn-collagen binding (Fig. 7). NMR analysis has revealed that residues G_778_-G_799_ of the collagen I α_1_(I) peptide can bind to structurally relaxed multimodular Fn regions FnI_6_FnII_1-2_FnI_7_ and FnI_8-9_ (refs 35, 36). Our data suggest that this template comprising several Fn modules can be at least partially destroyed by stretching Fn fibrils. This conclusion is supported by complementary observations showing that the binding of bacterial adhesion peptides to a neighbouring region of Fn (FnI_1-5_, see Fig. 1) is regulated by stretch-induced changes in the distances between binding sites that prevent multimodular interactions and result in a lower binding strength^26^,^27^. If Fn stretching does not directly destroy collagen interaction with FnII_1-2,_ stretching may alter the distances between modules within either collagen binding region and/or between the two binding regions themselves. Indeed, the two collagen binding regions FnI_6_FnII_1-2_FnI_7_ and FnI_8-9_ were previously hypothesized to act synergistically^35^. The overall affinity might thus be regulated by separating the multivalent binding sites on Fn by stretching. Although the inner core of the FnI and FnII modules is stabilized by disulfide bridges^55^, the bonds are located such that the modules’ N- and C-termini can extend on fibre stretching while the secondary structure of their inner cores and of the putative collagen binding sites might only be slightly distorted^26^,^27^. Since, in cell culture, Fn fibres are most relaxed immediately after they are generated, but become progressively bundled and stretched over time^45^,^46^,^56^, our proposed mechanism also provides a possible explanation for the observation that collagen I assembly is dependent on active Fn fibrillogenesis, even in the presence of a pre-existing Fn matrix^11^,^28^.

The strain propagation and distribution through a network of ECM fibrils made of distinct structural components can thus be highly non-uniform and might cause age- or disease-dependent switches between outside-in integrin signalling. Here we show that the presence of mature collagen fibres reduced the strain exerted on Fn matrix fibres (Figs 3 and 4). Significantly, the strain reduction of Fn fibrils in maturing ECM could only occur if collagen/Fn interactions are maintained. Mature collagen fibres became the primary load-bearing elements thus impairing the progressive stretching of Fn associated with cell-mediated remodelling of Fn-only ECMs^45^,^46^. Fibroblasts might thus significantly stretch and unfold Fn fibrils in situations in which Fn is the predominant ECM component, such as in early stages of wound healing and development. In the later stages of these processes as well as in homoeostatic tissues, a more-relaxed Fn conformation might be stabilized by the presence of collagen fibres. Our current study illustrates that there can be an unequal distribution of cell-generated strains between the different structural components of complex ECMs and suggests that, in ECMs with high collagen content, Fn fibril tension could actually be decreased despite increases of other microenvironment properties (for example, rigidity). For example, tumour cell-conditioned media can cause stromal fibroblasts to increase Fn fibril strain in early Fn ECMs *in vitro*^57^. Yet in many cases, tumour stroma is enriched in collagen^2^,^3^,^8^ and exhibits an elevated rigidity^58^. Future studies and novel labelling approaches, such as the recently described dual-antibody-staining technique^59^, are needed to investigate whether Fn fibrils are indeed more relaxed in actual cancer stroma tissue, in which it is very challenging to incorporate FRET-labelled Fn.

When collagen matrix is absent, force-induced unfolding of Fn might not only activate binding sites, for example those inducing Fn-fibrillogenesis^17^,^19^, but may also destroy others^14^,^22^,^25^,^26^. Elevated and uncontrolled collagen deposition, as is often seen in fibrosis and cancer stromagenesis^5^,^8^, could adversely affect the normal operation and effects of Fn unfolding^14^,^60^. The altered composition of the ECM, in the presence versus the absence of collagen, could also affect cell signalling, for example, by altering cells’ integrin usage. Cells use different integrins to bind to Fn and collagen I (ref. 61), which can differentially regulate intercellular signalling and cell phenotype^62^,^63^,^64^,^65^,^66^. Interestingly, it was recently shown that binding of heparin—an ECM molecule that is upregulated during inflammation—can regulate Fn conformation^59^.

Over the past decades, the growing realization that cell-matrix interactions are mechano-regulated has fundamentally changed our basic understanding of how the microenvironment determines cell fate. Here we provide the first example, to our knowledge, of mechanical forces also tuning the interactions between different structural ECM components. The cell-mediated remodelling of a Fn matrix into a predominantly collagen matrix is a common theme in development and wound healing. Our findings therefore directly change our understanding of how normal tissue formation—essential for tissue engineering and regenerative medicine—and its pathological aberrations (for example, cancer stromagenesis and fibrotic diseases) might occur. The mechano-regulation of structural ECM component interactions among each other and their associated matrix architectures provides another as yet unrecognized paradigm of how forces can impact the mechanobiology of matrix and thereby cell and tissue fate.

## Methods

### Fn isolation and fluorescent labelling

Fn was isolated from human plasma (Zürcher Blutspendedienst SRK, Switzerland) as previously described^23^. Briefly, 2 mM phenylmethylsulphonyl fluoride and 10 mM EDTA were added to human plasma and spun at 15 × 10^3^*g* for 40 min. Fn was isolated from the treated plasma via gelatin-sepharose chromatography (Pharmacia) and eluted with 6 M urea. Purity was assessed by silver stain and western blot. Isolated Fn was stored at −80 °C in 6 M urea.

Fn was doubly labelled with Alexa Fluor 488 (donor) on amines and Alexa Fluor 546 (acceptor) on free sulfhydryls (Molecular Probes, Invitrogen) as previously described^23^. Briefly, isolated Fn in urea was further denatured by adding an equal volume of 8 M guanidine HCl and then incubated for 1 h with a 20-fold molar excess of Alexa 546 maleimide. The Fn was then separated from unreacted dye and transferred into an amine labelling buffer (PBS with 0.1 M NaHCO_3_, pH 8.5) by size-exclusion chromatography (PD-10 Sephadex column, Amersham). The elutant was incubated with a 65-fold molar excess of Alexa 488 succinimidyl ester for 1 h, then separated from the unreacted dye and transfered to PBS using a PD-10 column. For specific experiments, Fn was singly labelled on amines with Alexa 488 succinimidyl ester or on free cysteines with Alexa 647 maleimide (Molecular Probes, Invitrogen) following similar procedures. Fluorescently labelled Fn was stored at −20 °C until needed. Labelled Fn was always used with an excess of 90% unlabelled Fn to prevent intermolecular energy transfer between molecules within a fibre^23^. Labelled and unlabelled Fn aliquots were centrifuged for 10 min. at 10,000*g* before use to remove aggregates.

Intramolecular FRET is expressed in terms of an intensity ratio, which is the peak acceptor intensity divided by the peak donor intensity. The correlation between the FRET ratio and solution conformation was determined by measuring the FRET ratio of Fn-FRET dissolved in different concentrations of guanidine HCl (Supplementary Table 1) as previously described^23^. Briefly, small channels were made between a microscope slide and coverslip using double-stick tape. The coverslip and slide were coated with 4% bovine serum albumin (BSA; Sigma-Aldrich) for 1 h, then rinsed with water and dried before assembly to prevent Fn-FRET binding to chamber surfaces. Fn-FRET at 0.1–0.2 g l^−1^ with or without 0–4 M GdnHCl or 50 mM DTT in PBS (Sigma-Aldrich) was then drawn into the chambers by capillary forces. Fn-FRET in solution within the channels was imaged through the coverslip using confocal microscopy as described, below. Cell culture and matrix manipulations

NIH 3T3 fibroblasts (American Type Culture Collection, ATCC) were cultured in DMEM (ATCC) supplemented with 10% newborn calf serum (Gibco, Invitrogen) in a humidified incubator at 37 °C with 5% CO_2_. Culture medium used in all experiments additionally contained 1% penicillin-streptomycin-fungizone (Gibco, Invitrogen).

Cells were seeded at a density of 60 × 10^3^ cells−cm^−2^ into eight-well chambered coverglasses (Lab-Tek, Nalge Nunc) that had been pre-adsorbed with 20 μg ml^−1^ unlabelled Fn for 30 min. The cells were allowed to attach for 30 min., then the medium was replaced with medium containing 50 μg ml^−1^ Fn and, if needed, 50 μg ml^−1^ ascorbic acid. Depending on the experiment, 10% of the added Fn was Fn-FRET or singly labelled Fn.

For the collagenase digestion experiments, live 3-day cultures were incubated with ∼50 CDU ml^−1^ bacterial collagenase type III (Sigma-Aldrich) in serum-containing growth medium for 3 h in the incubator. The samples were then washed with PBS and fixed for immunostaining.

R1R2 inhibition experiments: The R1R2 peptide has been previously described^51^,^52^ and was manufactured by GenScript. The amino acid sequence was as follows: GLNGENQKEPEQGERGEAGPPLSGLSGNNQGRPSLPGLNGENQKEPEQGERGEAGPP. R1R2 was added at a concentration of 2.5 μM directly to the cell culture medium in addition to ascorbic acid and Fn. Cells were cultured for 3 days in the presence of R1R2 before immunostaining to confirm collagen I deposition and imaging to measure FRET. Control experiments to detect a potential collagen-independent effect of R1R2 on FRET (Supplementary Fig. 2) were performed by decellularizing 3-day, ascorbic-acid-supplemented ECMs (0.5% Triton-X 100, 20 mM NH_4_OH), and then measuring FRET before and after incubating with 25 μM R1R2 for 2 h at 37 °C.

### Immunostaining

Samples were fixed with 4% formaldehyde for 30 min. and blocked with 5% donkey serum and 2% BSA for 1 h. The samples were incubated with a 1:100 dilution of rabbit-anti-mouse collagen I polyclonal antibody (Chemicon, Millipore) in 1% donkey serum for 1 h. They were then incubated with a 1:200 dilution of 647H-donkey-anti-rabbit secondary antibody (FluoProbes; 653/675 ex/em) in PBS with 5% donkey serum for 1 h. Samples were kept in PBS at 5 °C and imaged within 24 h.

### Confocal microscopy and FRET analysis

Z-stack images were acquired with an Olympus FV1000 confocal microscope with an oil-immersion 1.35 numerical aperture × 60 objective. Our basic acquisition procedures for FRET imaging have been described previously^23^. Briefly, donor and acceptor intensities were detected simultaneously with two photomultiplier tubes (PMTs). Variable Band-pass Filters were set to restrict the detected wavelengths to 515–525 nm and 567–577 nm for the donor and acceptor channels, respectively. Excitation settings were varied slightly between experiments to minimize photobleaching while maximizing signal-to-noise ratio. Each image was the average of three scans, calculated internally by the microscope software (Kalman filtering). Immunostained collagen in a field-of-view was independently imaged after the FRET image had been acquired.

The acceptor and donor images were analysed using MATLAB (MathWorks) as previously described^23^. Briefly, after correcting for the background noise and differences in the scaling of the PMTs, the acceptor intensities were divided, pixel-by-pixel, by the donor intensities to yield the FRET ratio. The FRET ratios were then compiled in histograms and also used to colour-code the fluorescence images.

For the co-localization analysis, the intensity of the 647 FluorProbes secondary antibody (collagen) was acquired in addition to the FRET channels. A custom MATLAB program was used to create plots of the normalized protein intensity versus FRET ratio per pixel. The collagen signal was normalized to account for differences in local Fn fibre density by dividing it, pixel-by-pixel, by the sum of the donor and acceptor intensities under 488 nm excitation^22^. The resulting curves of collagen/Fn versus binned FRET ratio (for example, Fig. 6e) were normalized to the total area under the curve to enable direct comparisons between data sets. Medians of normalized collagen/Fn values in each bin were used for analyses in the inset in Fig. 6e and in Supplementary Fig. 3.

### CLP binding to single Fn fibres

Arrays of trenches with widths from 20 to 50 μm, lengths of greater than 400 μm, and a depth of 20 μm were produced following previously published protocols^22^,^67^. Briefly, PDMS replicates were produced by pouring degassed PDMS over a patterned Si wafer (1:10, curing agent to polymer). After curing for 4 h at 80 °C, the PDMS master replica was peeled from the Si master. The microstructures were then cast into a thin film of PDMS (20–30 μm) on top of 0.25-mm-thick PDMS sheets (Specialty Manufacturing, Saginaw, MI) that were cut into rectangles of 5 × 1.5 cm (ref. 22). To facilitate the covalent attachment of the Fn fibres to the upper plateau region of the PDMS sheets, the PDMS structures were treated with air plasma (0.1 mbar for 60 s), functionalized with amino groups (3% 3-aminopropyltriethoxysilane in water) for 15 min and coupled with reactive groups by incubation with 1% glutaraldehyde (Sigma-Aldrich) in water for 30 min. The surfaces were rinsed with water, dried and mounted in a custom-built strain device^22^.

Single Fn fibres were generated as previously described^19^,^22^. Briefly, drops of 300 μg ml^−1^ Fn solution (10% Fn-Alexa647, 90% unlabelled) were deposited on the PDMS sheets. Fibres were drawn out of the droplet and deposited across the PDMS trenches with a pipette tip (Fig. 7a). The sample was rinsed and kept hydrated with PBS. Covalent bonds formed between the glutaraldehyde-functionalized PDMS surface and the lysine residues on the Fn fibres positioned on the plateau regions between the trenches. Since deposited Fn fibres are under residual stress after the deposition onto the PDMS substrate (∼140% strain), the fibres were deposited onto pre-strained PDMS sheets in a custom strain device and subsequently either relaxed or further stretched^22^. The macroscopic strain of the silicone sheet has been shown to be equivalent to the strain experienced by fibres on the sheet^23^.

CLP, a synthetic, single-strained collagen-derived peptide covering residues Q_778_-G_799_ (ref. 35) with a terminal cysteine used for photolabelling, was synthesized by Genescript Corporation, NJ, USA. The amino acid sequence is as follows: QRGVVGLOGQRGERGFOGLOGC^35^. A 200 μg ml^−1^ solution of the CLP was labelled with Alexa Fluor 488 (Molecular Probes, Invitrogen) as described above and stored at −20 °C until needed.

To quantify the CLP binding to the fibrillar Fn of specific conformation, manually deposited fibres were prepared and incubated with 4% BSA for 30 min. After rinsing with PBS, the samples were incubated with CLP-Alexa488 for 30 min. The samples were rinsed with PBS and imaged in PBS. CLP-Alexa488 and Fn-Alexa647 intensities were simultaneously detected as described above. The data were analysed as previously described^25^. Briefly, after subtracting the background, the CLP-Alexa488 intensity was divided by the Fn-Alexa647 intensity, pixel-by-pixel. Values in Fig. 7d were normalized to the mean ratio at the 140% strain point (the strain at which the fibres are initially deposited).

## Additional information

**How to cite this article:** Kubow, K. E. *et al.* Mechanical forces regulate the interactions of fibronectin and collagen I in extracellular matrix. *Nat. Commun.* 6:8026 doi: 10.1038/ncomms9026 (2015).

## Supplementary Material

Supplementary Information Supplementary Figures 1-3, Supplementary Table 1 and Supplementary References
