# Supplementary material for: Mechanical forces regulate the interactions of fibronectin and collagen I in extracellular matrix
Source: Nat Commun. 2015 Aug 14;6:8026. doi: 10.1038/ncomms9026 (PMC4539566; doi:10.1038/ncomms9026)
Supplement: Supplementary Information — Supplementary Figures 1-3, Supplementary Table 1 and Supplementary References [file ncomms9026-s1.pdf]

Supplementary Figures

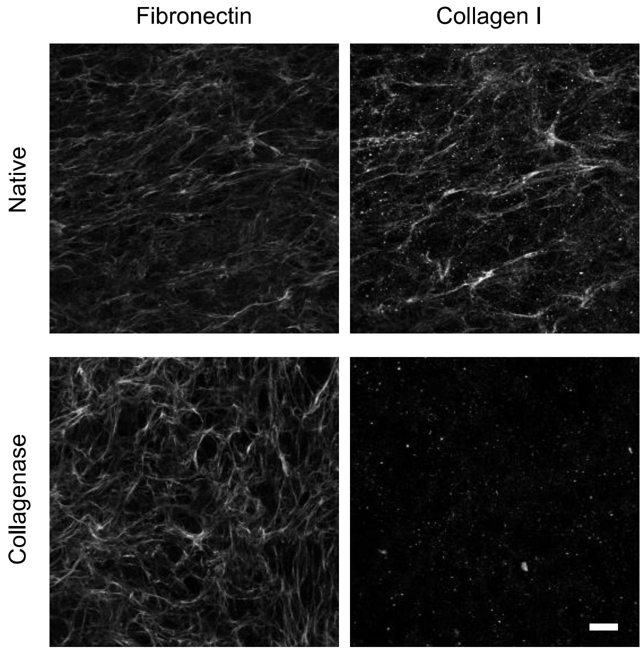

Supplementary Figure 1: Treating ECMs with collagenase removes collagen I but not Fn. Supplement to Fig. 4. Live, Fn-FRET-labeled, three-day samples were cultured in the presence of ascorbic acid were digested with collagenase for 3 h or kept un-digested ('native') as in Fig. 4. The samples were subsequently fixed and immunostained for collagen I. The figure shows images from native and digested samples that are representative of results from three independent experiments. Collagenase treatment removes collagen I, but does not eliminate Fn. Note that the native and collagenase images are from separate samples.

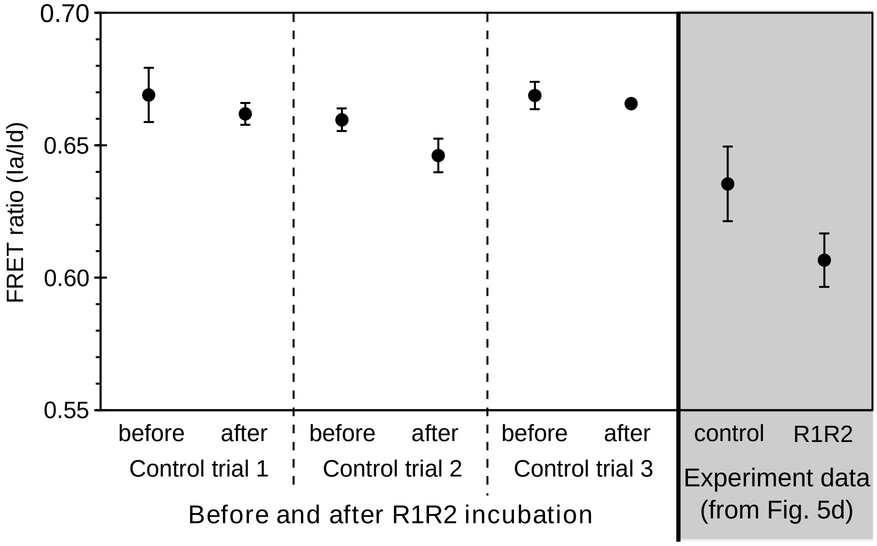

Supplementary Figure 2: Effect of R1R2 on the FRET ratio of Fn ECMs without collagen. Supplement to Fig. 5. Control experiments to determine whether R1R2 has a direct effect on FRET ratios, independent of its effect on collagen-Fn binding. FRET measurements of de-cellularized three-day ECMs, grown in the absence of ascorbic acid, were performed before and after a 2 h incubation with the R1R2 peptide under the same conditions as in Fig. 5. De-cellularized matrices were used so that paired before and after measurements could be performed on the same samples without interference from progressive unfolding caused by cell contractility[1] (note that there is no indication that R1R2 directly elevates cell contractility[2]). The first three sections of the plot show the mean FRET ratios before and after R1R2 treatment for three independent experiments (five measurements for each experiment). There is no significant change in mean FRET ratio caused by incubation with R1R2 in the absence of collagen ($p = 0.12$, paired t-test). For comparison, the left-most section (gray) shows the mean FRET ratios from matrices containing collagen that were treated with or without R1R2 (these data are identical to those in Fig. 5d).

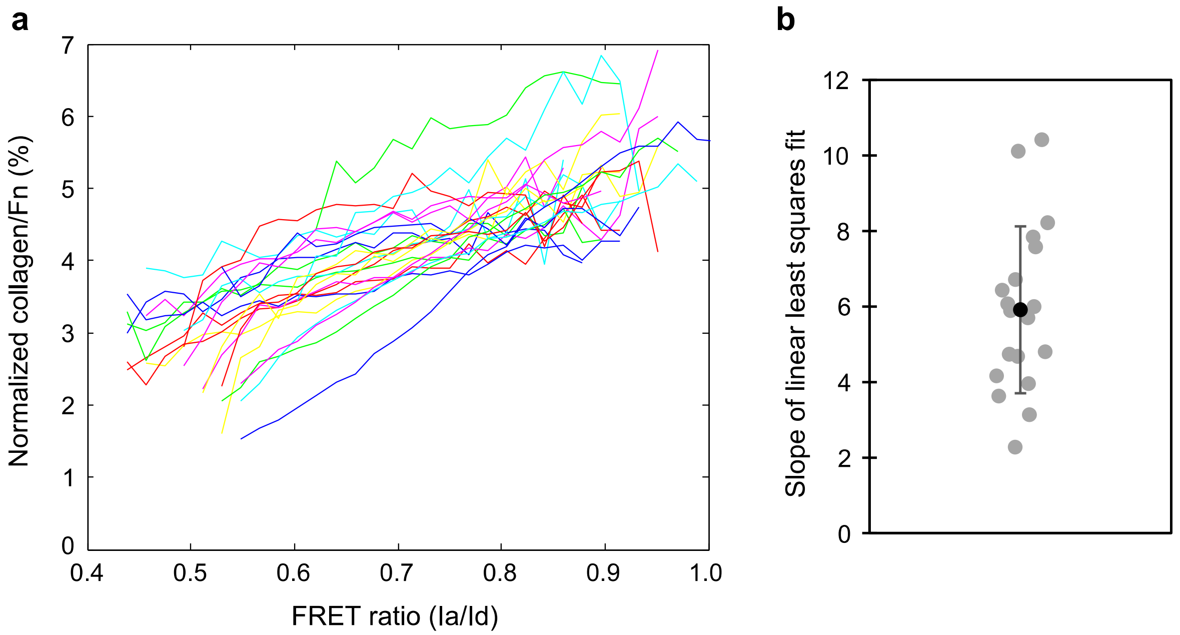

Supplementary Figure 3: Co-localization analysis of collagen to a FRET Fn matrix.

Supplement to Fig. 6. **(a)** Traces of median collagen/Fn intensity ratios vs. FRET ratios (plotted as in Fig. 6e) for 23 measurements from four experiments. Each trace has been normalized to the area under the curve and expressed as a percent. **(b)** Traces in (a) were fit to a linear model using least squares regression. The fitted slopes of the 19 traces (out of 23) that had R-squared values > 0.7 are plotted as individual gray points. All slopes are significantly nonzero, $p < 0.01$, as indicated by their 99% confidence intervals (not shown). The mean (+/- S.D.) slope value is also plotted (black).

Supplementary Table 1: FRET ratios as a function of denaturant concentration for different batches of Fn-FRET in solution. The denaturation curves for the different batches of Fn-FRET used in this study vary slightly due to differences in labeling efficiencies.

	FRET ratios* of Fn-FRET in different concentrations of GdnHCl		
Applicable Figures	0 M	1 M ‡	4 M
3, 4	1.14 +/- 0.057	0.697 +/- 0.047	0.574 +/- 0.012
4	1.14 +/- 0.069	0.711 +/- 0.053	0.573 +/- 0.048
5	1.02 +/- 0.001	0.560 +/- 0.002	0.462 +/- 0.002
5	0.854 +/- 0.075	0.598 +/- 0.035	0.490 +/- 0.019
5, S2	0.955 +/- 0.002	0.526 +/- 0.001	0.442 +/- 0.001

* Mean +/- SEM of three independent measurements; each row represents a different batch of Fn-FRET.
‡ The 1 M values were measured with monomeric Fn to eliminate intramolecular FRET due to cross-over of the dimer arms[3].

Supplementary References

1. Antia, M., Baneyx, G., Kubow, K. E. & Vogel, V. Fibronectin in aging extracellular matrix fibrils is progressively unfolded by cells and elicits an enhanced rigidity response. Faraday Discussions 139, 229-249 (2008).
2. Sottile, J. et al. Fibronectin-dependent collagen I deposition modulates the cell response to fibronectin. American Journal Of Physiology-Cell Physiology 293, C1934-C1946 (2007).
3. Smith, M. L. et al. Force-induced unfolding of fibronectin in the extracellular matrix of living cells. PLoS Biol 5, e268 (2007).
